# Pulmonary Lymphangitic Spread of Multiple Myeloma as Early Relapse after Autologous Stem Cell Transplantation

**DOI:** 10.1155/2021/5590975

**Published:** 2021-04-03

**Authors:** Bicky Thapa, Gulrayz Ahmed, Meera Mohan, Volodymyr Shponka, Parameswaran Hari

**Affiliations:** ^1^Division of Hematology and Oncology, Department of Medicine, Medical College of Wisconsin, Milwaukee, WI, USA; ^2^Division of Pathology and Laboratory Medicine, Department of Medicine, Medical College of Wisconsin, Milwaukee, WI, USA

## Abstract

Clinical relapses early after autologous stem cell transplantation portrays an inferior clinical outcome. Early relapse in this setting with extramedullary disease (EMD) of lung involvement in multiple myeloma is rare. To our knowledge, this is the first reported case of lymphangitic spread of myeloma with pulmonary parenchymal and pleural involvement occurring at first relapse.

## 1. Introduction

Multiple myeloma (MM) is characterized by malignant clonal proliferation of plasma cells and is known for relapsing disease course with a small cure fraction. Extramedullary disease (EMD) in MM exemplifies a clonally evolved population of myeloma cells that can survive independent of the bone marrow (BM) microenvironment and confers treatment resistance and adverse clinical outcome. Herein, we report a rare case of fulminant pulmonary lymphangitic relapse of myeloma with extramedullary pulmonary involvement as the first clinical presentation of relapse occurring within 90 days after an upfront autologous stem cell transplantation (ASCT).

## 2. Case

**Figure 1:**
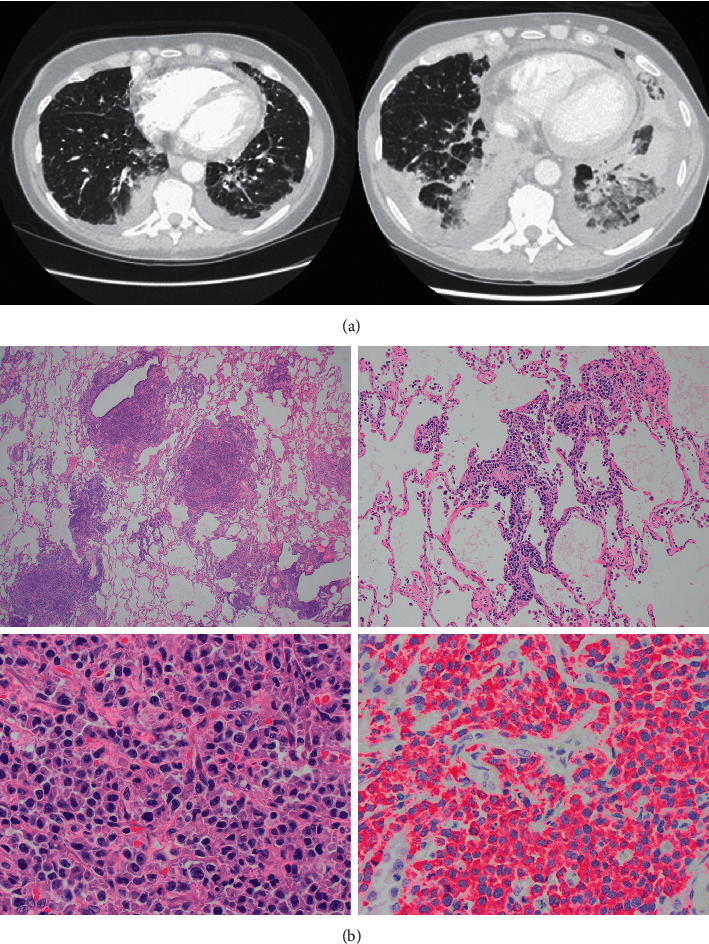
(a) Coronal sections of computed tomography showing worsening of bilateral lower lobe consolidation along with extensive ground-glass density, interlobular septal thickening. (b) Pathological specimen showing infiltration of lung parenchyma by kappa light chain restricted atypical plasma cells with a large, round to irregular nuclei, variably prominent nucleoli, and moderate amounts of eosinophilic cytoplasm. Flow cytometry showed 17.9% plasma cells with the following immunophenotypic markers (CD5 (−), CD10 (−), CD19 (−), CD20 (−), CD38 (bright+), CD45 (dim+), CD56 (partial dim+), CD138 (variably+), and CD319 (+)) (Hematoxylin and eosin; 20*x*, 100*x*, 500*x*, and 500*x*, respectively.)

A 63-year-old female presented with R-ISS stage II IgG kappa MM after disease progression from monoclonal gammopathy of undetermined significance (MGUS). She had high-risk cytogenetics del17p (unknown clone size) on FISH with complex karyotype. She presented with back pain resulting from a thoracic vertebral paramedullary mass at T12 with no neurological compromise. ^18^F-FDG PET at diagnosis showed multiple focal lesions with no extramedullary disease. The patient proceeded with 30 Gy local radiation to thoracic spine and induction therapy with KRD for 4 cycles achieving a partial response (IMWG criteria), followed by high dose chemotherapy (HDC) and ASCT. ASCT did not further deepen the response and the patient attained partial response, hence the plan was to proceed with additional KRD consolidation followed by maintenance therapy. However, the patient presented with worsening shortness of breath (SOB) and cough approximately D + 90 after ASCT requiring hospitalization. A computed tomography (CT) at admission showed new bilateral lower lobe infiltrates with consolidation and innumerous pulmonary nodules (Figure 1). She was started on broad-spectrum antibiotics for suspected bacterial pneumonia. Incidentally, she was also found to have worsening pancytopenia with an elevated lactate dehydrogenase 609 unit/L without laboratory evidence of hemolysis. Peripheral smear reported 1% circulating plasma cells by morphology. An extensive infectious workup was unrevealing. Bronchoscopy with bronchoalveolar lavage (BAL) revealed atypical plasma cells with a malignant pleural effusion. A repeat CT scan was performed for persistent hypoxia, which showed further worsening of bilateral lower lobe consolidation along with extensive ground-glass density, interlobular septal thickening, and patchy consolidation throughout the upper lobes as well as superior segments of the lower lobes (Figure 1). Video-assisted thoracoscopy and lung biopsy showed extensive patchy involvement of lung parenchyma by clonal kappa-restricted plasma cells in perivascular, peribronchial, and periseptal distribution in addition to focally forming variably sized aggregates (Figure 1). Bone marrow (BM) biopsy was 95% cellular, with 88.9% monotypic kappa-restricted plasma cells. FISH studies on the BM aspirate showed deletion of 1p with a gain of 1q, a gain of FGFR3 (4p16), the gain of MYC (8q24), the gain of CCND1 (11q13), monosomy 13, a complex *t* (14; 16) IGH-MAF translocation, TP53 deletion, and gain of MAFb (20q12) observed in 29–96% of plasma-cell-enriched cells analyzed. This is in contrast to the FISH studies at initial diagnosis which showed 1q21 gain, the gain of MYC, *t* (14; 16) in addition to the original TP53 clone. Cytogenetics showed a complex tetraploid karyotype with (del)17P. Taken together, the clinical picture was consistent with a marrow relapse and extramedullary myelomatous lung involvement with lymphangitic spread and malignant pleural effusion. The patient became progressively hypoxic, requiring admission to the intensive care unit. She was started on methylprednisolone 1 gram daily for 3 days followed by bendamustine (90 mg/m^2^) × 2 days. Unfortunately, the MM was proved difficult to control, thus we proceeded with salvage KPACE with minimal symptomatic improvement. Given the incurable nature of disease and limited prognosis, the patient opted for hospice care.

## 3. Discussion

The incidence of EMD in MM had increased from 6.5% in 2005 to 23.7% in 2014 based on a recent registry study, and this is ascribed to the improving survival of MM patients and routine integration of novel imaging techniques such as PET CT and MRI DWIBS in clinical practice [1]. It has been postulated that repeated relapse after exposure to novel agents promote a clonal evolution and establishment of more aggressive clones, which account for an increase in EMD, but this theory had been refuted by more recent studies [2]. As expected, EMD is more frequent in the relapsed refractory setting with distribution showing mostly a predominance of liver involvement in the posttransplant setting compared to more skin and soft tissue at diagnosis [3]. EMD in general portends poor prognosis due to the aggressive nature of the disease and resistance to chemotherapy. While EMD is rare at the initial diagnosis, it confers an inferior 5-year progression free survival (PFS) of 21% overall survival (OS) of 31%^3^.

EMD correlates with a high-risk 70-gene expression profiling signature with enrichment of MF molecular subgroup and the PR molecular subgroup [3]. In addition, EMD at the time of diagnosis is a robust predictor of EMD in the relapse setting [4]. It had been postulated that EMD develops because of a “bone marrow escape” of more clonally evolved plasma cells capable of surviving independently of bone marrow microenvironment akin to primary plasma cell leukemia (pCL). Indeed, whole-exome sequencing and gene expression profiling analysis of pCL showed enrichment for high-risk mutations in addition to downregulation of adhesion molecules present in the extracellular matrix supporting their extramedullary survival [5, 6]. TP53 deletions and MYC overexpression by FISH were shown to be presented in a significant proportion of patients presenting with EMD like our patient driving a state of increased proliferation and decreased apoptosis analogous to double-hit lymphoma. Furthermore, discordance in the cytogenetic markers between the bone marrow and EMD has also been observed in keeping with the clonal evolution of these populations [7, 8]. Cytogenetic or FISH analyses were not performed in the lung EMD in our patient, and the genetics is unknown. Concomitant secondary central nervous system involvement has a distinctive association with EMD and spinal fluid examination in our patient was unremarkable.

Our case is unique due to the clinical presentation of early fulminant relapse within 90 days of ASCT with lymphangitic and parenchymal involvement of the lung along with the pleura. The relapse pattern could be due to a hematogenous inoculum of plasma cells in the lung followed by perivascular lymphatic spread into pulmonary parenchyma. In addition, early relapse within the first 24 months of ASCT occurring in 35–38% of newly diagnosed multiple myeloma (NDMM) confers an inferior clinical outcome [9]. More recently, a study reported a unique subset (12.9%) of NDMM relapsing within 12 months of ASCT. Cytogenetics defined high risk and ultra-high risk was significantly greater in this subset of patients with a progression free survival of 18 months and overall survival of 26 months, once again underscoring the dismal outcome in this subset of patients [10, 11].

Our case highlights the very aggressive nature of the disease depicted by the presence of high-risk cytogenetics of (del)17p at initial diagnosis, suboptimal response to induction therapy, early relapse within 3 months of ASCT with additional high-risk cytogenetics including 1q21, and complex tetraploid karyotype with poor response to salvage therapy. Interestingly, in our case, there is a striking discordance in the flow cytometry expression of CD56 or neural cell adhesion molecule (NCAM) with BM being negative and extramedullary lung site positive perhaps suggesting a clonal evolution [12]. Published case reports of EMD with lung involvement demonstrated high mortality with dismal prognosis [13–17] (Table 1). So far, 11 cases have been published in the last 15 years with pulmonary parenchymal EMD. Majority of these described cases presented with lung EMD at the time of diagnosis while only two cases had lung EMD after ASCT (one within 2 months of ASCT and the other 4 years after ASCT). Overall clinical outcome has been dismal (Table 1). Clinical presentation is variable and a high index of suspicion is crucial [13–17, 23]. Besides, our patient was perplexing with COVID-19-related pneumonia high on the differential. Due to rarity and underrepresentation in clinical trials, there is no consensus on the optimal management of these subsets of patients; we opted for salvage multiagent chemotherapy in combination with novel agents. Long-term favorable outcomes have been described following alloSCT [24], which option our patient declined to pursue. Indeed, the expression of CD 38 is preserved in the BM and EMD possibly implying the role of novel CD 38 targeting antibodies, bispecific T-cell engager (BiTE), and chimeric antigen receptor (CAR) T-cell therapy. Extramedullary involvement of lung parenchyma with a lymphangitic spread as the first evidence of an early relapse after an upfront ASCT is extremely rare and associated with poor outcomes.

## Figures and Tables

**Table 1:** Summary of case reports with myelomatous lung involvement and their outcomes.

Case reports	Age and gender	Diagnostic modality	Subtype of myeloma	Cytogenetics	EMD involvement of the lung	Treatments	Status
Marmor et al. [13]	65, F	Lung biopsy	IgG kappa	NA	At initial diagnosis of MM	None	Expired

Nitu et al. [14]	60, M	BAL	IgG	NA	At initial diagnosis of MM	None	Unknown

Yuan et al. [15]	58, M	BAL	IgG kappa	*t* (11; 14) (q13; q32) and del 13q14.3	4 years after ASCT	Induction: vincristine, adriamycin, and dexamethasone (VAD) followed by ASCT while hospitalized: thalidomide, dexamethasone, bortezomib, and doxorubicin.	Expired

Kushwaha et al. [18]	60, M	Lung biopsy and BMBx	IgG	NA	At initial diagnosis of MM	None	Expired

Sahin Balcik et al. [18]	62, M	Lung biopsy	IgA kappa	Del(13q), hypodiploidy	At initial diagnosis of MM	Vincristine, adriamycin, dexamethasone (VAD)	Expired

Ravinet et al. [17]	61, M	Lung tissue autopsy	IgG kappa	*t* (4; 14)	6 months after diagnosis of MM; EMD lung within 2 months of ASCT	Induction: bortezomib, thalidomide, and dexamethasone followed by ASCT	Expired

Lok et al. [16]	64, M	Chest wall mass biopsy and BAL	Lambda light chain	NA	5 months after diagnosis of MM	NA	Expired

Rai et al. [19]	55, F	CT guided lung biopsy, BMBx	NR	NA	At initial diagnosis of MM	NA	NR

Abhishek et al. [20]	58, M	Lung biopsy	NR	NA	1 year after diagnosis of MM	Induction therapy: bortezomib, thalidomide, and dexamethasone. Treatment for relapse not reported.	NR

Shah et al. [21]	60, M	CT guided FNAC of lung mass, BMBx	IgG	NA	At initial diagnosis of MM	Melphalan, prednisone	Unknown

Furuncuoglu et al. [22]	42, M	CT guided lung biopsy	Lambda light chain	NA	At initial diagnosis of MM	NA	Unknown

NA, not available; NR, not reported; BAL, bronchoalveolar lavage; BMBx, bone marrow biopsy; ASCT, autologous stem cell transplant; CT, computed tomography; EMD, extramedullary disease.

## Abbreviations

KRD: Kyprolis (carfilzomib), Revlimid (lenalidomide), and low-dose dexamethasone
KPACE: Kyprolis (carfilzomib) 20 mg·m^2^ D1,2 and 36 mg/m^2^ D8, 9, cisplatin 10 mg/m^2^ D1-4, adriamycin 10 mg/m^2^ D1-4, cyclophosphamide 400 mg/m^2^ D1-4, and etoposide 40 mg/m^2^ D1-4^18^F-FDG
PET: ^18^F-2-Fluoro-2-deoxyglucose positron emission tomography/computed tomography
FISH: Fluorescence in situ hybridization
MRI DWIBS: Diffusion-weighted whole-body imaging with background body signal suppression
IMWG: International myeloma working group.
